# From Dizziness to Dysphagia: A Complex Presentation of Lateral Medullary Syndrome

**DOI:** 10.7759/cureus.73118

**Published:** 2024-11-06

**Authors:** Isabella Canut, Bilal Farooqui, Joshua Hickman

**Affiliations:** 1 Neurology, Philadelphia College of Osteopathic Medicine, Suwanee, USA; 2 Internal Medicine, AdventHealth New Smyrna Beach, New Smyrna Beach, USA

**Keywords:** dysphagia, lateral medullary syndrome, pathophysiology, prognosis, stroke, vertigo, wallenberg’s syndrome

## Abstract

Lateral medullary syndrome (LMS), also known as Wallenberg syndrome, is a rare neurological condition most commonly resulting from occlusion of the posterior inferior cerebellar artery (PICA). This syndrome is characterized by a constellation of symptoms including vertigo, ataxia, sensory deficits, and cranial nerve abnormalities, which arise due to infarction of the lateral medulla. We report the case of a 74-year-old female patient with a history of hypertension who presented to the emergency department with acute onset of vertigo and an unsteady gait. Following a diagnosis of LMS confirmed with imaging of the brain, the patient's hospital course was further complicated with the onset of atrial fibrillation and dysphagia, necessitating gastrostomy tube placement. Despite the challenges, early intervention and multidisciplinary care helped stabilize her condition.

## Introduction

Lateral medullary syndrome (LMS) is a rare condition resulting from ischemia of the posterior inferior cerebellar artery (PICA), a branch of the vertebral artery, which leads to infarct of the lateral medulla. Gaspard Vieussux first identified the condition in 1808, and was then later comprehensively described by Adolf Wallenberg between 1895 and 1901 [1].

LMS manifests through a distinct constellation of neurological deficits. These symptoms include hemisensory disturbance (ipsilateral face, contralateral body), ipsilateral cerebellar signs, and ipsilateral Horner’s syndrome (ptosis, miosis, and anhidrosis). The loss of pain and temperature sensation on the contralateral side of the body is due to involvement of the spinothalamic tract [1,2]. Damage to the spinal trigeminal nucleus results in impaired pain sensation on the ipsilateral side of the face. Additionally, the syndrome is characterized by dysphagia and dysarthria due to involvement of the nucleus ambiguus, and ipsilateral ataxia from cerebellar or inferior cerebellar peduncle damage [2]. The presence of vertigo and nystagmus is indicative of vestibular nuclei involvement, while disruption of the hypothalamospinal fibers leads to the classic ipsilateral Horner’s syndrome [1,3]. 

LMS is the most prevalent form of posterior ischemic stroke. Each year, approximately 800,000 individuals in the United States experience acute strokes, with 83% classified as ischemic. Of these ischemic strokes, 20% affect the posterior circulation. Assuming nearly half of these cases involve Wallenberg syndrome, it can be estimated that over 60,000 new cases of LMS occur annually in the United States. LMS typically affects men in their sixth decade [4].

## Case presentation

A 74-year-old female patient with a history of poorly controlled type 2 diabetes mellitus, hypertension, hyperlipidemia, and deep vein thrombosis presented to the emergency department (ED) with acute dizziness, nausea, and a left-sided temporal headache. She reported experiencing a similar episode one week prior, characterized by acute dizziness and nausea triggered by positional changes. These symptoms lasted approximately five minutes and then resolved with rest. In the ED, the patient was unable to ambulate and had a left-sided unsteadiness. She denied chest pain, fevers, chills, visual disturbances, or focal neurological deficits.

Upon arrival, she was afebrile (98.2°F/36.8°C) with a blood pressure of 161/91 mmHg, and described experiencing a positional vertigo. She also had a left-sided facial droop which her husband mentioned was a chronic finding. Physical examination revealed left-beating nystagmus on rightward gaze, a left pronator drift, and gait instability with a leftward drift. Electrocardiogram (ECG) demonstrated sinus rhythm, and a chest X-ray was unremarkable. Initial laboratory findings indicated anion gap metabolic acidosis, hyperglycemia, and mild diabetic ketoacidosis (Table 1). Urinalysis indicated a urinary tract infection (Table 2).

**Table 1:** Blood tests on initial presentation

Test	Result	Reference Range
Sodium	141 mEq/L	135-145 mEq/L
Potassium	3.5 mEq/L	3.5-5.0 mEq/L
Chloride	102 mEq/L	95-105 mEq/L
Anion Gap	17 mEq/L	4-12 mEq/L
Urea Nitrogen	17.0 mg/dL	8-20 mg/dL
Serum Creatinine	0.66 mg/dL	.60-1.30 mg/dL
Glucose	253 mg/dL	70-99 mg/dL
Calcium	9.2 mg/dL	9-10.5 mg/dL
Beta-hydroxybutyrate	1.06 mmol/L	Less than .5 mmol/L

**Table 2:** Urinalysis on initial presentation

Test	Result	Reference Range
Color	Colorless	Colorless
Clarity	Clear	Clear
Leukocyte esterase	2+	Negative
Nitrite	1+	Negative
pH	5.5	5-9
Blood	1+	Negative
Ketones	1+	Negative
Glucose	4+	Negative

**Figure 1:**
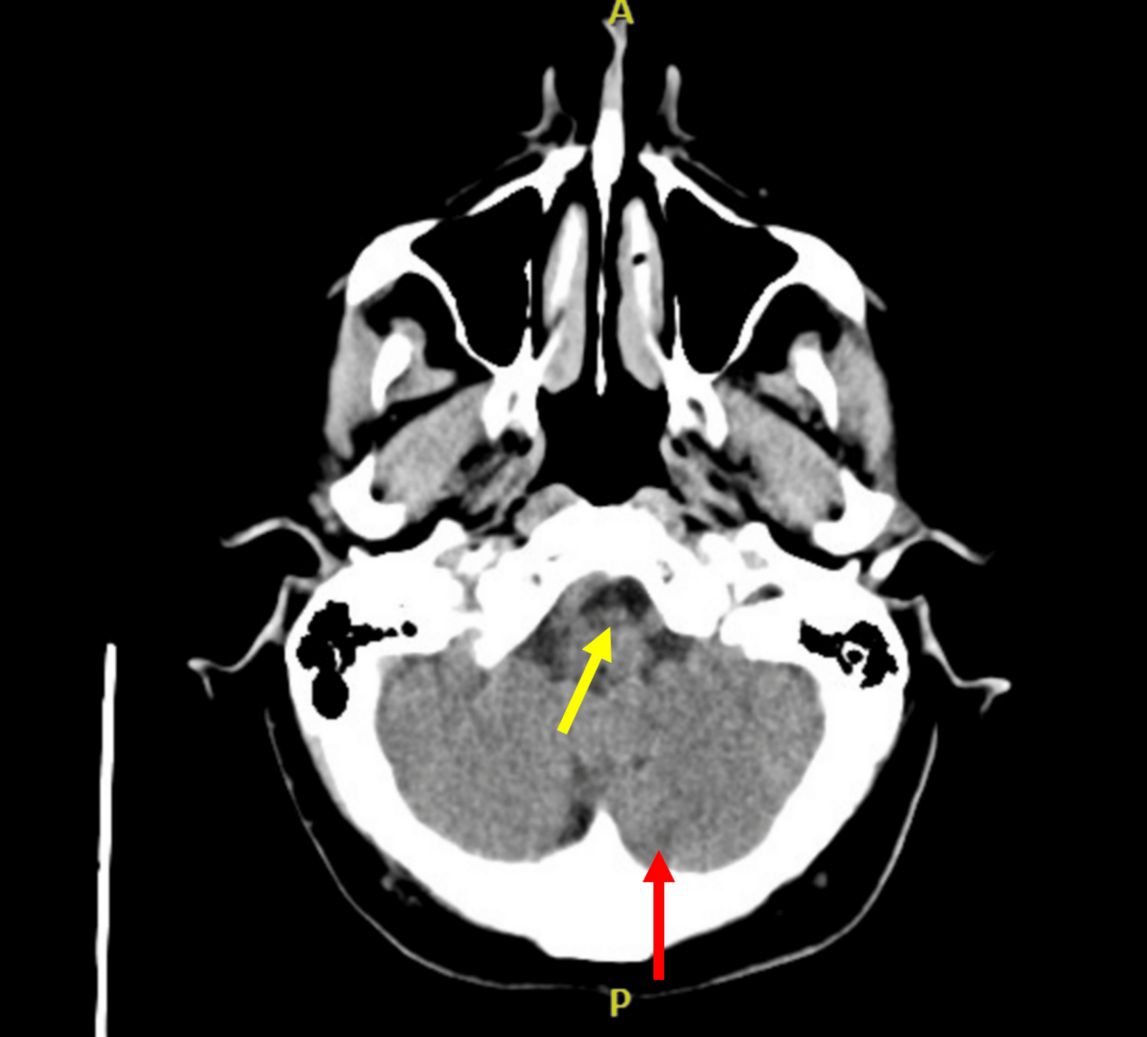
CTA head taken on day of admission showing focal severe stenosis of the left A2 segment, moderate stenosis of the left distal P1 segment, and a severe stenosis of the proximal left P3 segment (red arrow), and a 2 mm basilar tip aneurysm (yellow arrow) CTA: computerized tomography angiopathy

**Figure 2:**
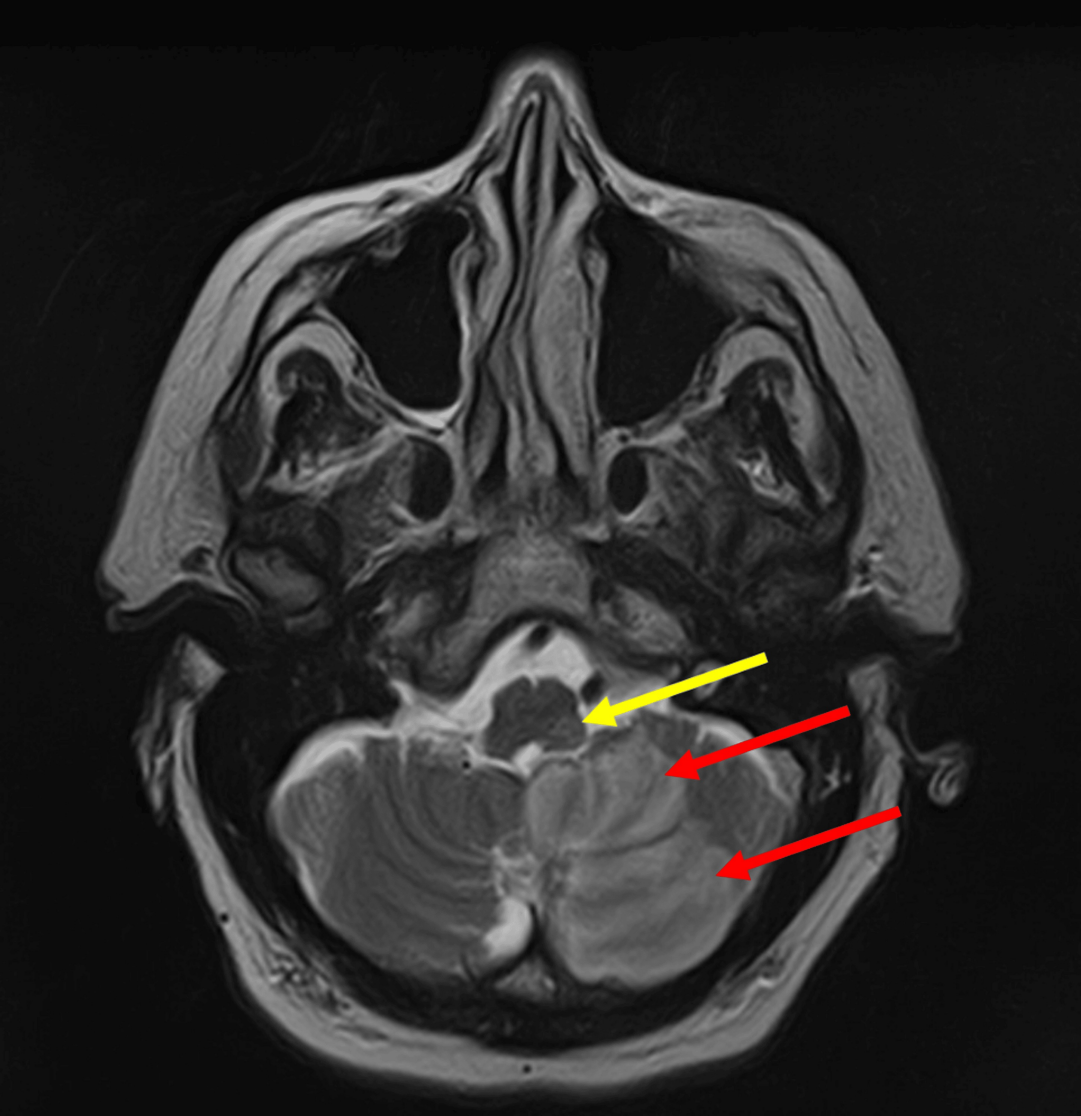
MRI of the brain taken on the second day of admission showing left PICA territory diffusion restriction (red arrows) with T2/FLAIR hyperintensity compatible with acute ischemic infarct, and diffusion restriction focus in the left dorsolateral aspect of the medulla oblongata (yellow arrow). No intracranial hemorrhage is seen. MRI: magnetic resonance imaging, PICA: posterior inferior cerebellar artery; FLAIR: fluid-attenuated inversion recovery

Non-contrast computed tomography (CT) of the head revealed focal severe stenosis of the left A2 segment, moderate stenosis of the left distal P1 segment, severe stenosis of the proximal left P3 segment, and a 2 mm basilar tip aneurysm (Figure 1). Magnetic resonance imaging (MRI) of the brain performed the following day confirmed a left PICA territory infarction with diffusion restriction in the left dorsolateral medulla (Figure 2).

**Figure 3:**
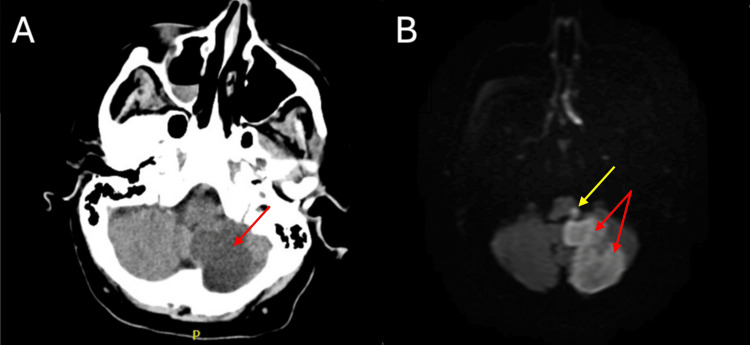
Repeat CT of the head (A, B) showing the previous infarct of the left cerebellum (red arrows) and left medulla (yellow arrow) without evidence of hemorrhagic transformation CT: computerized tomography

On the third day of admission, the patient developed atrial fibrillation with rapid ventricular response, requiring diltiazem for rhythm control, and anticoagulation with heparin post-clearance by neurology. In addition, nursing staff reported concerns for possible aspiration, and subsequent chest X-ray revealed a left lower lobe infiltrate consistent with aspiration pneumonia. The patient developed leukocytosis, meeting the criteria for sepsis, and her antibiotic regimen was escalated to piperacillin-tazobactam. Due to significant dysphagia, confirmed by two failed swallow studies showing variable laryngeal penetration and aspiration with nectar-thick liquids, honey-thick liquids, and pudding, a gastrostomy tube was placed. A repeat CT scan showed a stable infarct without hemorrhagic transformation (Figure 3). The gastrostomy tube was removed the following day due to an improvement in her swallowing function.

The patient showed clinical improvement over the course of her hospitalization which lasted six days. Her discharge plan included long-term anticoagulation with Eliquis, physical and occupational therapy, rehabilitation, and outpatient follow-up with neurology and cardiology.

## Discussion

LMS arises from ischemia in the vascular territory of the PICA, resulting in characteristic neurological signs. This case highlights the importance of maintaining a high index of suspicion for LMS in elderly patients presenting with non-specific symptoms such as vertigo, dizziness, and gait disturbances, particularly when vascular comorbidities such as hypertension and diabetes are present. In our patient, the combination of her acute presentation and multiple vascular risk factors calls attention to the multifaceted nature of stroke pathophysiology.

Role of the HINTS (head impulse, nystagmus, and test of skew) exam

The HINTS exam is a bedside test that assesses eye movements to help differentiate peripheral from central causes of vertigo, especially in acute settings. Typical findings of LMS, which is a central lesion, include a normal head impulse test and direction-changing nystagmus, which are suggestive of a central etiology.

Risk factors

Hypertension is the most common risk factor for LMS, followed by smoking and diabetes mellitus. Other etiologies include vertebral artery dissection, which can occur secondary to neck manipulation or head trauma, as well as connective tissue disorders such as Marfan syndrome, Ehlers-Danlos, and fibromuscular dysplasia. In younger patients, vertebral artery dissection is the leading cause of LMS. Embolic stroke originating from the heart is another important etiology to consider, especially in patients with underlying arrhythmias, cardiac dysfunction, or valvular heart disease [5]. Large artery atherothrombosis is responsible for approximately 75% of cases, with cardioembolism accounting for 17%, and vertebral artery dissection contributing 8% [4]. 

Vascular risk factors, particularly hypertension, diabetes, and hyperlipidemia played a crucial role in this patient’s condition. Hypertension accelerates atherosclerosis, leading to the occlusion of arteries such as PICA, which can contribute to stroke [4,5]. In the current patient, long-standing hypertension and hyperlipidemia likely contributed to the atherothrombotic changes leading to infarction. The previous episode of atrial fibrillation, which occurred during hospitalization, further complicated the clinical picture. Anticoagulation and heart rate control were critical in her management.

Differential diagnosis

The initial presentation of vertigo, dizziness, and headache in an elderly female patient raises a broad differential ranging from peripheral causes of vertigo such as benign positional vertigo, vestibular neuritis, or Meniere’s disease, to central causes such as posterior circulation strokes [6], as highlighted in this case. Differentiating LMS from other causes of dizziness is crucial as misdiagnosis can delay life-saving interventions. In this patient, the combination of neurological signs and symptoms (nystagmus, pronator drift, and gait instability) directed the care team toward neuroimaging and diagnosis of LMS. 

Imaging

Diagnosing LMS is usually based on clinical examination as well as patient history. MRI with diffusion-weighted imaging (DWI) is the preferred method for confirming an infarction in the inferior cerebellar region or lateral medulla. However, up to 30% of patients with non-disabling strokes may not show a lesion on DWI, classifying them as DWI-negative stroke cases [4]. As seen in this case, a brain MRI confirmed the left PICA territory infarct. While non-contrast CT scans are often the first imaging modality, MRI provides better sensitivity for early ischemic changes where CT findings can be subtle or even absent [7-9].

Management

Treatment of the patient involved addressing the underlying cause of infarct, preventing a secondary stroke, and managing complications [7]. Anticoagulation with heparin was initiated in response to atrial fibrillation, and the patient was started on antihypertensives to control blood pressure. The development of dysphagia necessitated gastrostomy tube placement due to the risk of aspiration. Following hospitalization, the long-term treatment of the patient’s comorbidities is best managed with a multidisciplinary approach: lifestyle modifications, anticoagulation, lipid-lowering therapy, rehabilitation treatment, as well as diabetes education to reduce future stroke risk [10-12]. 

Prognosis

The prognosis for patients with LMS is generally favorable in terms of functional recovery [6,7]. Patients often recover and resume previous activities with improved respiratory care. However, mortality can occur in the acute phase due to complications such as aspiration pneumonia, and fatal outcomes related to sleep apnea have also been documented in several cases [7]. Early intervention with rehabilitation can help patients recover motor and sensory function. Dysphagia however can have a significant impact on quality of life and requires long-term management such as speech therapy and nutritional support. Continued monitoring for respiratory issues is crucial not only in the acute phase but also long-term follow-ups [6-8]. 

## Conclusions

This case highlights the importance of considering LMS in the differential diagnosis of a patient presenting with vertigo and gait disturbances, especially in patients with known vascular risk factors such as hypertension. This case is unusual in that there were no sensory disturbances as well as the development of dysphagia later on during the course of hospitalization. Early recognition and prompt neuroimaging are crucial for diagnosis and management. While LMS is rare, timely intervention can improve outcomes through stroke prevention and rehabilitation. Further research is needed to optimize therapeutic strategies for this condition and improve functional outcomes for affected patients.
